# Sphingomyelin Synthase 1 Is Essential for Male Fertility in Mice

**DOI:** 10.1371/journal.pone.0164298

**Published:** 2016-10-27

**Authors:** Anke Wittmann, Marcus O. W. Grimm, Harry Scherthan, Marion Horsch, Johannes Beckers, Helmut Fuchs, Valerie Gailus-Durner, Martin Hrabě de Angelis, Steven J. Ford, Neal C. Burton, Daniel Razansky, Dietrich Trümbach, Michaela Aichler, Axel Karl Walch, Julia Calzada-Wack, Frauke Neff, Wolfgang Wurst, Tobias Hartmann, Thomas Floss

**Affiliations:** 1 Helmholtz Zentrum München, Institute of Developmental Genetics, Ingolstädter Landstrasse 1, 85764 Neuherberg, Germany; 2 Saarland University, Experimentelle Neurologie, 66424 Homburg/Saar; Germany; 3 Institut für Radiobiologie der Bundeswehr in Verb. mit der Univ. Ulm, 80937 Munich, Germany; 4 Helmholtz Zentrum München, German Mouse Clinic, Institute of Experimental Genetics, Ingolstädter Landstrasse 1, 85764 Neuherberg, Germany; 5 Helmholtz Zentrum München, Research Unit Analytical Pathology, Institute of Pathology, Ingolstädter Landstraße 1, 85764 Neuherberg, Germany; 6 Helmholtz Zentrum München, German Mouse Clinic, Institute of Pathology, Ingolstädter Landstrasse 1, 85764 Neuherberg, Germany; 7 Technische Universität München, Co Helmholtz-Zentrum München; 8 Deutsches Zentrum für Neurodegenerative Erkrankungen e.V. (DZNE), Site Munich, Schillerstrasse 44, 80336 München, Germany; 9 Max-Planck-Institute of Psychiatry, Kraepelinstr. 2–10, 80804 München, Germany; 10 Institute for Biological and Medical Imaging, Helmholtz Zentrum München and Technische Universität München, 85764 Neuherberg, Germany; Universite Blaise Pascal, FRANCE

## Abstract

Sphingolipids and the derived gangliosides have critical functions in spermatogenesis, thus mutations in genes involved in sphingolipid biogenesis are often associated with male infertility. We have generated a transgenic mouse line carrying an insertion in the sphingomyelin synthase gene *Sms1*, the enzyme which generates sphingomyelin species in the Golgi apparatus. We describe the spermatogenesis defect of *Sms1*^-/-^ mice, which is characterized by sloughing of spermatocytes and spermatids, causing progressive infertility of male homozygotes. Lipid profiling revealed a reduction in several long chain unsaturated phosphatidylcholins, lysophosphatidylcholins and sphingolipids in the testes of mutants. Multi-Spectral Optoacoustic Tomography indicated blood-testis barrier dysfunction. A supplementary diet of the essential omega-3 docosahexaenoic acid and eicosapentaenoic acid diminished germ cell sloughing from the seminiferous epithelium and restored spermatogenesis and fertility in 50% of previously infertile mutants. Our findings indicate that SMS1 has a wider than anticipated role in testis polyunsaturated fatty acid homeostasis and for male fertility.

## Introduction

Sphingolipids are common components of mammalian cell membranes [1]. Sphingomyelin synthase 1 (SMS1/SGMS1) and the isoenzyme SMS2 both catalyze the *de novo* synthesis of sphingomyelin (SM) and diacylglycerol (DAG) from ceramide (Cer) and phosphatidylcholine (PC), while the paralog sphingomyelin synthase-related (SMSr) has no SM synthase activity [2–4]. Several tissue-specific isoforms of SMS1, produced from different promoters as well as through alternative splicing, give rise to two different proteins, of which only one has enzymatic function [5,6]. SMS1 is located in the cis-Golgi apparatus and responsible for the bulk production of SM [2,3,7,8]. However, due to vesicle-mediated trafficking between the Golgi apparatus and the plasma membrane, the highest concentration of SM and other complex sphingolipids (SL) is found in the outer leaflet of the plasma membrane [9]. Here, the diverse SL species have various functions in structure, adhesion and signaling. At the plasma membrane, these functions are mediated by SMS2, which unlike SMS1 plays a major role in controlling Cer levels at the plasma membrane [1,10,11]. However, sphingolipids have similar intracellular functions in all cellular compartments, including the nucleus [12] and an imbalance in lipid composition can lead to disease.

In testis, SM composition is characterized by an unusual high content of long chain (LC), e.g. docosahexaenoic acid (DHA), very long chain (VLC) and hydroxylated (2-OH) omega-3 (n-3) and omega-6 (n-6) poly unsaturated fatty acids (LC-/VLC-/PUFA). In men and mice n-3 and n-6 fatty acids (FAs) cannot be generated *de novo*, but strictly depend on dietary consumption. However, shorter n-3/6 FAs can be converted to longer n-3/6 PUFA, which is assumed to represent the source for VLC-PUFA-SM. Highest levels of testicular SM-PUFA, n-VLC-PUFAs and 2-OH-VLC-PUFAs is a consequence of Ceramide synthase 3 (CerS3) expression in pachytene spermatocytes and spermatid stages ([13]; [14]). Several mutations in enzymes of the SL metabolism caused severely impaired fertility [15–21] underlining their importance for spermatogenesis. Especially for SMS1 knock-out male mice absence of viable progeny has been reported [15]. The reasons for this remained unknown to date.

In our study, we analysed an insertional mutation in the anabolic enzyme SMS1 and its consequences for male fertility. Indeed, infertility was observed in 66% of *Sms1* homozygous mutant adult males. Infertility was caused by sloughing of meiotic and spermatid stages and their subsequent accumulation in the epididymides. Lipid profiling of testes revealed a decrease of LC-PUFAs in *Sms1*^-/-^ animals. Male infertility was ameliorated by supplementary diet of DHA and eicosapentaenic acid (EPA), indicating an essential role for SMS1 in the supply with n-3 unsaturated FA substrates.

## Materials and Methods

### Sms1-/- animals

All animal experiments were carried out in accordance with the European Communities Council Directive (20/0/63/EU). Animal experiments, which included complementation of food by PUFA and subsequent imaging using fluorescent dyes, were performed according to an allowance by the District Government of Bavaria (number TVA 189–12) to TF from 06/18/2013 after previous reviewing and ethical commission of the Bavarian Government on 04/10/2013 according to §15 paragraph 1 and §8 paragraph 1 of the German Animal Protection law. All surgery was performed under sodium pentobarbital anesthesia, and all efforts were made to minimize suffering. Proper animal caretaking was provided by the Helmholtz Center animal facility (AVM) according to standard rules. Animals were group housed in ventilated standard cages on a 12-hour light-dark cycle and had ad libitum access to food and water. The mutant *Sms1* allele (MGI: *Sms1*^*Gt(E201D11)Wrst*^, in the following referred to as *Sms1*^-/-^) was generated by gene trap technology as described previously [22]. Males were backcrossed six times to C57Bl/6J mice (Charles River Laboratories) and *SMS1*^*+/+*^ and *Sms1* mutant littermates were used in this study. Genotyping was done by triplet-PCR amplification using the following primers: *Tmem23*_fw (5’-gccggaaattaaaaagaacaatga-3’), *Tmem23*_rv (5’-gcaatgcagggtgcttcttcc-3’) and Spli_rv2 (5’-gccaaacctacaggtggggtcttt-3’. A band of 577bp represents the mutant fragment and 999bp represents the wild-type fragment. All PCR fragments were sequence-verified initially. PCR cycling settings were 95°C, 5’–(94°C, 1’–60°C, 1’–72°C, 1’) x 35–72°C, 7’ for all reactions.

### Organ preparation for Protein- and RNA-based analysis

Animals were sacrificed using CO_2_. Tail clips were taken for regenotyping. Organs were dissected, kept on dry ice and subsequently stored at -80°C until further investigations.

### DNA isolation

Genomic DNA was isolated from tail tips by incubation with 500μl of Lysis-Buffer (10mM KCl, 20mM Tris, 0.1% Non.N40, 0.1% Tween) and 20μg of Proteinase-K per tail tip at 60°C for at least 3h. For Proteinase-K inactivation samples were heated to 95°C for 10’ prior to centrifugation and separation of the supernatant.

### Protein isolation

Organ samples were manually homogenized in 500μl ice cold RIPA-Buffer (50mM Tris-HCl (pH7.5), 150mM NaCl, 1% Triton X-100, 0.5% Sodiumdeoxycholat, 0.1% Sodiumdodecylsufat, 2mM EDTA (pH8.0), with added Complete Protease Inhibitor Cocktail (Roche), immediately after organ removal. After sonication RIPA-soluble and insoluble protein fractions were separated by centrifugation (15000g, 20’, 4°C) and stored at -80°C.

### Assessment of protein concentrations

Concentration of RIPA-soluble protein was measured by the bicinchoninic acid (BCA) Protein Assay Reagent (Thermo scientific) according to the manufacturer’s instructions. Briefly, 25μl of BSA standards, ranging from 25μg/μl to 2mg/μl, and sample dilutions were applied to a 96 well flat bottom plate and incubated for 30’ at 37°C with 200μl of BCA working reagent (CuSO_4_/bicinchoninic acid (1:50) per well. Readout of the colorimetric assay was performed at 562nm.

### Western Blot

40ng of RIPA-soluble protein per sample was mixed with 5μl NuPAGE® LDS Sample Buffer (4x; Invitrogen) and 0.2μl β-Mercaptoethanol and was heated to 95°C for 3’. For SDS-PAGE protein samples and 10μl of SeeBlue® Plus2 Pre-Stained Standard (Invitrogen) were applied to a NuPAGE® Novex Bis-Tris 4–12% Gel (Invitrogen) for 1h at 200V, RT. Proteins were transferred to PVDF membranes (Immobilon-P, Immobilon® Transfer Membranes, Millipore) for 1.5h at 30V, RT. The membrane was blocked with 5% skim milk (Sigma, in TBS-T (0.05%) for 1h, RT and subsequently incubated with primary antibodies at 4°C, overnight. After washing with 0.05% TBS-T, the secondary antibody was applied to the membrane in a 1:10000 dilution for 1h, RT. After 3 x 10’ in 0.05% TBS-T, the blot was developed using Hyperfilm ECL and the ECL detection reagents (Amersham).

Quantification of the protein signal, was performed using ImageJ and statistical analysis of signal intensity was done using the student’s T-test. Statistical significance was considered at * p ≤ 0.05, **p ≤ 0.01 and *** p ≤ 0.001.

### RNA isolation

1ml Trizol was added per sample. Samples were homogenized and incubated for 5’ at RT prior to centrifugation at 15000g for 10’ at 4°C. The supernatant was mixed with 200μl of chloroform, incubated for 8’ at RT and centrifugated at 15000g for 15’ at 4°C. The supernatant was mixed with 530μl of isopropanol and incubated at -20°C, overnight. Remaining RNA-isolation steps were carried out using the RNeasy Kit (Qiagen) according to the manufacturer’s instructions, including DNAse treatment. RNA was stored at -80°C until further processing.

### Northern Blot

20μg per RNA sample were mixed with 15μl of RNA loading dye (Thermo scientific) and heated to 50°C for 30’ before the samples were loaded to a 1.2% formaldehyde- agarose (FA)-gel. Gel electrophoresis took place in FA-running buffer (FA-gel buffer (200mM MOPS, 50mM sodium acetate, 10mM EDTA, pH 7.0) with added 12.3M formaldehyde) at 100V for 3h. RNA integrity was checked by visualization of the 18S and 28S band. The gel was washed in dH_2_O and two changes of 10x SSC (1.5M NaCl, 150mM Sodiumcitrate), 15’ each. The RNA was blotted overnight on an Amersham Hybond^TM^-N^+^ membrane (GE-Healthcare) using 10x SSC, subsequently UV-crosslinked and pre-hybridized in Church Buffer (1M Na_2_HPO_4_, 1M NAH_2_PO_4_, 1% BSA, 7% SDS, 1mM EDTA (pH 8.0), 0.1mg/ml ssDNA (Sigma), pH 7.4) at 65°C for 1h, to prevent unspecific binding.

For Northern Blot probes 801bp of *Sms1* exon 7 were PCR-amplified using the primers Sms1_e7fw (5’–GCGAACGAATGTTTGGACACCG– 3’) and Sms1_e7rv (5’–GCAGCCACTGAAATAGCCAGAGT– 3’) and cloned for multiplication. Exon 7 is at least partially present in all four protein coding transcripts. A *Gapdh* probe was PCR-amplified and cloned using the primer pair of Gapdh_fw (5’–TCTCCGCCCCTTCTGCCGATG– 3’) and Gapdh_rv (5’–CAGCCCCGGCATCGAAGGTG– 3’ and served as a loading control. Probes were P^32^-labeled using the Rediprime II random labeling System (Amersham) and diluted in Church Buffer to 1,500 counts/μl (*Sms1* probe) and 1,000 counts/μl (*Gapdh* probe). After blocking, the membrane was incubated with labeled cDNA-hybridization-probes at 65°C, overnight. Redundant radioactivity was removed by washing with 2x SSC (RT, 2x 10’), 2x SSC-1% SDS (RT, 10’), 1x SSC-1% SDS (65°C, 10’). Kodak X-ray films were applied overnight. Statistical analysis was performed using the student’s T-test. Statistical significance was considered at * p ≤ 0.05, **p ≤ 0.01 and *** p ≤ 0.001.

### Histological analysis and organ pathology

Histological analysis and organ pathology was performed within the German Mouse Clinic as described [23]. A total of 46 mice of the *Sms1* mouse line at different ages were analysed for morphological changes.

Tails were stored at -70°C for genetic reconfirmation. Body weight, body length and weight of internal organs such as heart, spleen and liver were determined. All organs were fixed in 4% buffered formalin and embedded in paraffin, 4μm sections were stained with hematoxylin and eosin (H&E) for histological examination. Periodic acid shiff (PAS) staining was applied for visualization of glycolipids. Images of tissue sections were acquired with an automated slidescanner (NanoZoomer-NDP-Hamamatsu, Germany). All slides were independently reviewed and interpreted by 2 pathologists, experienced in mouse pathology. Statistical analysis was done using the Student’s T-test, statistical significance was considered at p value (<0,05).

### Immunohistochemistry (IHC)

Analysis of proteins, which are differentially expressed during spermatogenesis was performed using IHC. IHC was carried out on 8μm sections of paraffin-embedded mouse testes and epididymides, using the streptavidin-peroxidase method, etiher with an automated immunostainer (DISCOVERY®XT Roche) or using the following protocol. Paraffin sections were rehydrated and subjected to heat-mediated antigen retrieval using Citrate-Buffer (DCS, ChromoLine). The slides were incubated with blocking-solution (TBS-T (0.1%), 1% BSA) for 25’, RT. Avidin- and Biotin-blocking was performed for 15’, RT, each (Avidin/Biotin Blocking System, Convance Signet Antibodies). Primary antibodies were applied (overnight, 4°C). Incubation with the secondary antibodies (30’, RT) was followed by DAB staining including 30’ incubation with Streptavidin Peroxidase (KPL) and application of 3,3’-diaminobenzidine (DAB) as substrate (DAB two components kit, DCS ChromoLine). Counterstaining was done using haematoxylin. (For specific information on primary and secondary antibodies see S1 Table)

### Transmission electron microscopy

Animals (*Sms1*^-/-^ n = 2, *SMS1*^*+/+*^ n = 2, age: 12 weeks) were transcardially perfused with 50ml ice cold PBS, prior to perfusion with 70ml of fixing solution (2.5% PFA, 2.5% glutaraldehyde in PBS). Testes and epididymides were removed and cut into 1mm^3^ cubes, which were post fixed in 2.5% glutaraldehyde containing 0.1M sodium cacodylate buffer (pH 7.4) at 4°C over night.

Tissues were fixed in 2.5% electron microscopy grade glutaraldehyde in 0.1 M sodium cacodylate buffer pH 7.4 (Science Services, Munich, Germany), postfixed in 2% aqueous osmium tetraoxide *(*Dalton, *1955*), dehydrated in gradual ethanol (30–100%) and propylene oxide, embedded in Epon (Merck, Darmstadt, Germany) and cured for 24 hours at 60°C. Semithin sections were cut and stained with toluidine blue. Ultrathin sections of 50 nm were collected onto 200 mesh copper grids, stained with uranyl acetate and lead citrate before examination by transmission electron microscopy (Zeiss Libra 120 Plus, Carl Zeiss NTS GmbH, Oberkochen, Germany). Pictures were acquired using a Slow Scan CCD-camera and iTEM software (Olympus Soft Imaging Solutions, Münster, Germany).

### In-vivo Optoacoustic Imaging of BTB Permeability

Animals were placed under isoflurane anesthesia and were imaged using a multispectral optoacoustic tomography (MSOT) system described previously [24]. In brief, mice were shaved using depilation cream around the testes region for imaging, and then placed in a prone position in the MSOT imaging system. Ultrasound coupling gel and water were used to provide acoustically-matched coupling between the tissue and acoustic detector array. The animal was positioned in the detector array to provide tomographic slices in the region of the testes. Tissue was illuminated over a range of wavelengths (700, 730, 760, 800, and 860 nm) and the resulting optoacoustic responses were used to reconstruct cross-sectional images through the testes using a back-projection algorithm [25]. During imaging, 2mg/kg bodyweight Indocyanine Green (ICG) was injected via the tail vein and serial multispectral images were acquired following injection. Finally, multispectral unmixing was done using linear regression [26,27] to derive the ICG absorbance signal. Region of interest (ROI) analysis of the unmixed signal provided the quantitative time course of ICG distribution and clearance within the testes with a time resolution of ~ 5 seconds. Quantification of the ICG absorbance signal was determined by taking the mean pixel intensity value of the ICG signal within the region of the testes.

### Lipid profiling

#### a. Testes preparation

Testes were dissected and homogenized in H_2_O on ice using a PotterS (Braun, Melsungen, Germany) at 1500 revolutions per minute and 50 strokes. Homogenates were adjusted to Protein amount of 10mg/ml with H_2_O, snapfrozen in liquid nitrogen and stored at -80°C for further experiments.

#### b. Protein amount determination

Protein determination was performed according to Smith et al. [28]. Briefly, we used a standard curve of bovine serum albumin with a concentration range of 0.1–1.2 μg/μl. 20μl of the standard solutions was pipetted onto a 96-well plate (Nunc, Langenselbold, Germany) and further 1–2μl of each sample was pipetted in triplicates onto the 96-well plate. 200μl reagent buffer, consisting of CuSO_4_/bicinchoninic acid (1:39; v/v) was added to each well using a multichannel pipette (Eppendorf, Germany). The plate was incubated for 15’ at 37°C followed by incubation at RT for 15’ while shaking (IKA, Staufen, Germany) at 300 revolutions per minute. Absorbance was measured using a MultiscanEX (Thermo Fisher Scientific, Schwerte, Germany) at a wavelength of 550nm.

#### c. Mass spectrometry

All measurements were performed using a 4000 quadrupole linear-ion trap (QTrap) mass spectrometer equipped with a Turbo Spray ion source (AB SCIEX, Darmstadt, Germany): The QTrap was connected to a Agilent Cap HPLC comprising of 1200 Capillary Pump, 1200 Micro Wellplate Sampler and 1200 Thermostat Module (Agilent, Böblingen, Germany). Data analysis was carried out using Analyst 1.5 software (AB SCIEX, Darmstadt, Germany). For determination of phosphatidylcholine, phospatidylcholine-plasmalogen, lyso-phosphatidylcholine, sphingomyelin and ceramide levels 20μl sample was injected into sample loop with the following running solvent gradient (0.0–2.4min, 30μl; 2.4–3.0min, 200μl; 3.0min, 30μl). Mass spectrometry settings and composition of running solvents used is specified below.

#### e. Measurement of diacylphosphatidylcholine (PCaa), phospatidylcholine-plasmalogen (PCae), lyso-phosphatidylcholine (lysoPC) and sphingomyelin (SM) levels

Measurement was performed according to Grimm et al. [29]. Briefly, 10μl of homogenates was applied onto a solvinert 96well plate with a 0.45μm sterile filter at the bottom (Millipore, Schwalbach, Germany). Samples were further incubated in a 5% phenylisothiocyanate solution diluted in C_2_H_5_OH/H_2_O/pyridine (1:1:1; v/v/v) for 20’. Samples were extracted using 5mM ammonium acetate buffer (300μl) in methanol using a multichannel pipette (Eppendorf, Germany) into a 1ml 96well deep well plate (Nunc, Langenselbold, Germany) and further diluted with 600μl of 5mM ammonium acetate dissolved in CH_3_OH/H_2_O, which also served as the only running solvent.

#### g. Measurement of ceramide levels

Ceramide measurement was performed according to Gu et al. [30]. In brief, 50μl of each samples was mixed with 500μl CH_3_OH/H_2_O/12M HCl (95/5/0.5; v/v/v) and sonificated for 1’ at 4°C. Mixture was shaken for 10’ at RT at maximum speed (Multireax, Heidolph Instruments, Schwabach, Germany). After adding 300μl H_2_O and 500μl CHCl_3_ samples were shaken for another 10’ at RT. Samples were centrifuges at 5000rpm for 10’ and the lower phase was transferred to a new tube. 700μl CHCl_3_ was added to the remaining two phases and mixture was shaken for 10’ at RT at maximum speed. After another centrifugation step as described above, the two lipid containing phases were combined and dried in a vacuum concentrator. Finally, lipids were dissolved in 300μl CHCl_3_/CH_3_OH (95/5; v/v) and transferred into a 96-deep well plate (Nunc, Langenselbold, Germany). Prior to injection the samples were diluted with 600μl 5mM ammonium acetate dissolved in CH_3_OH and shaken at 300rpm (IKA, Staufen, Germany) for 10’. The running solvent for ceramide analyses was composed of CH_3_OH/ H_2_O (97/3; v/v).

#### h. Statistical analysis

All experiments were performed using at least five different animals per genotype and analysed in triplicates. Statistical significance was set at * p ≤ 0.05, **p ≤ 0.01 and *** p ≤ 0.001; n.s. = not significant and statistical analysis was determined by two-tailed Students t-test.

### TUNEL staining and quantification of apoptotic events

TUNEL staining was performed with the ApopTag® Fluorescein or Peroxidase *In Situ* Apoptosis Detection Kit (Millipore, California) according to the manufacturer’s instructions. Briefly, formalin fixed, paraffin embedded testes sections were deparaffinated, hydrated and treated with Proteinase K (20μg/ml). For fluorescent or peroxidase-mediated labeling, single/double strand brakes were prolonged by addition of dNTPs (labeled and unlabeled) via terminal deoxynucleotidyl transferase (TdT) for 1h at 37°C. The reaction was terminated and fluorescein-conjugated anti-digoxygenin antibody or anti-digoxigenin conjugate was applied for 30’, RT, respectively. Peroxidase-mediated staining required additional quenching of endogenous peroxidase after Proteinase K treatment and incubation with peroxidase substrate after applying of the conjugate. Sections were counterstained using DAPI (0.5μg/ml) or Haematoxylin and analysed by fluorescent or light microscopy, respectively.

TUNEL-positive cells in an average of 400 tubuli per animal (wild-type n = 3, *Sms1*^-/-^ n = 4) were counted for quantification of apoptotic events.

### PUFA (DHA/EPA) diet

For the dietary assessment 20 week old male *Sms1* mice were used for a fertility-test. For this, *Sms1* wildtype (n = 2) and *Sms1*^+/-^ (n = 4) males, serving as controls, and *Sms1*^-/-^ (n = 6) males were each provided with one eleven week old C57Bl/6J female. After 3 weeks these females were separated from the males and kept for additional 3 weeks. The litters obtained were recorded. Subsequently to separation of the first batch of females, *Sms1* males of all genotypes were provided with a second female each. In parallel, a mix of n-3 fatty acid ethylesters (Omega-3 90 EE) including 38% DHA and 46% EPA (kindly provided by KD Pharma (Bexbach)) was manually fed to the male *Sms1* mutants in an oral dose of 9mg (total n-3 fatty acids)/day/animal for 13 weeks.

## Results

### The *Sms1* gene trap mutation

**Fig 1:**
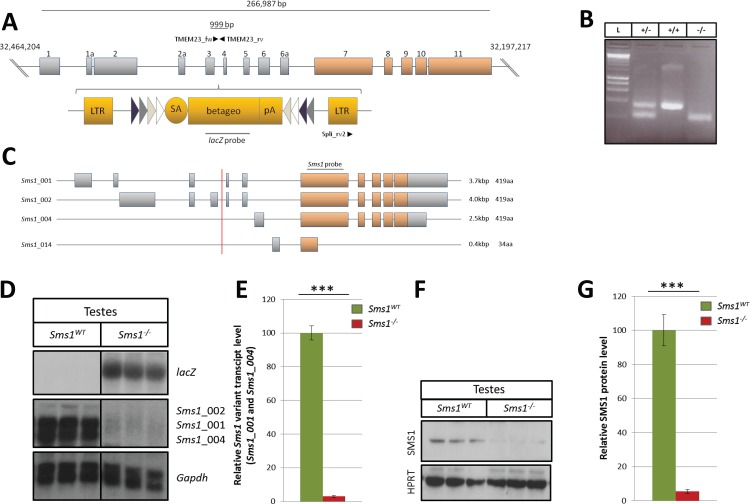
*The* β*-geo* gene trap cassette was inserted in *Sms1* intron 3 and leads to disruption of testes-specific transcripts and protein expression. (A) Schematic drawing of the β-geo gene trap cassette (yellow bars) and its insertion in intron 3 of the *Sms1* gene. Coding exons are indicated by brown colour. Genotyping primers (TMEM23_fw, TMEM23_rv and Spli_rv2) are indicated as black arrowheads. The location of the *lacZ* probe used for Northern blotting is depicted by a grey line. (B) Mice were genotyped by triplet-PCR. The wild-type allele: 999bp, mutant allele 577bp; Ladder (L), heterozygous (+/-), wild-type (+/+), homozygous (-/-). (C) Schematic illustration of the four protein coding splice variants. Red line illustrates the gene trap insertion site. *Sms1* specific Northern blot probe, (801bp), is indicated by a grey bar. (D) Northern blot of testes RNA revealed a reduction of testes specific (*Sms1*_001, _002, _004) *Sms1* splice variants in *Sms1*^-/-^ animals. (E) Quantification of the *Sms1*_001 and *Sms1*_004 splice variants in testes of *Sms1*^-/-^ in comparison to *Sms1*^*WT*^ animals. (F) Immunoblot analysis of testes tissue with HPRT as a loading control. (G) Quantification of testes SMS1 protein levels of *Sms1*^*WT*^ and *Sms1*^-/-^. *Sms1*^*WT*^ (n = 3), *Sms1*^-/-^ (n = 3). Data is shown as mean ± SEM, *p≤0.05, **p≤0.01, ***p≤0.001.

The *Sms1* gene comprises 14 exons, of which the upstream exons 1-6a are non-coding. The *Sms1* mutation described here is an insertion of a conditional gene trap vector in intron 3, which locates upstream of the first open reading frame in exon 7 (Fig 1A; MGI: Sgms1^Gt(E201D11)Wrst^). *Sms1*^+/-^ animals were interbred to homozygosity which was determined by triplet-PCR (Fig 1B). *Sms1*^-/-^ animals were obtained at Mendelian ratios, viable with no obvious phenotypes and had life expectancies of at least 24 months. When animals were further examined for subtle morphological alterations, we determined a slightly higher body weight, and a reduction in liver weights of male *Sms1*^-/-^, while body, heart and spleen weights were not significantly affected (S2 Table). SMS1 protein expression in testes samples was analysed using a polyclonal antibody (Santa Cruz). SMS1 protein was reduced to 5.5 ± 1.2% (p = 0.0005) in *Sms1*^-/-^ animals, as compared to protein levels in the wild-type (Fig 1F and 1G).

### Alternative splicing and alternative promoters in *Sms1*

*Sms1* is widely expressed in a number of tissues and organs [5]. Various transcripts have been described which are derived from either alternative splicing (*Sms1*-001) or alternative promoter activity (*Sms1*-002, *Sms1*-004 and *Sms1*-014; Fig 1C, [31]). Two alternative promoters in mouse *Sms1* are predicted downstream of the insertion based on exclusive use of the upstream non-coding exons 6 or 6a (*Sms1*-004 and *Sms1*-014; Fig 1C). In order to determine and quantify, which *Sms1* transcripts were expressed and which variants were disrupted by the intronic gene-trap insertion, we performed Northern blots using a probe covering 801bp of the first coding exon 7, which was at least partially present in all four protein coding transcripts of the gene (Fig 1C). The alternative transcripts *Sms1*-004 (2.5kb) and *Sms1*-001 (3.7kb) were the major residual transcripts in testes, showing a strong mean reduction to 3.1 ± 0.7% (p = 0.0001) as compared to wild-type levels, reflecting the detected amount of SMS1 protein (Fig 1D and 1E). The transcript *Sms1*-002 (4.0kb), which initiates upstream of the insertion site, was expressed in marginal amounts in wild-type but was not detected in testes *of Sms1*^-/-^ males. The short *Sms1*-014 transcript was neither detected in wild-type nor *Sms1*^-/-^ testes samples (Fig 1C and 1D).

### Lipid profiling of testis tissue

**Fig 2:**
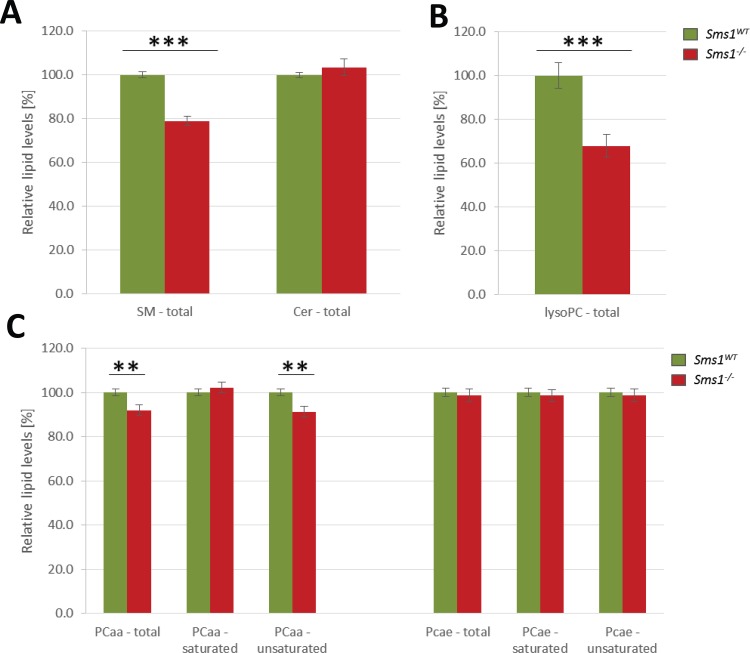
Lipid profiling of *Sms1*^-/-^ testes reveals reduction in sphingomyelin, lyso-phosphatidylcholine and unsaturated diacylphosphadidylcholins. (A-C) Relative lipid levels of (A) total sphingomyelin (SM) and ceramide (Cer). (B) Total lyso-phosphatidylcholine (lyso-PC) and (C) total diacylphosphatidylcholine (PCaa) and phosphatidylcholine-plasmalogen (PCae) levels. PCaa and PCae levels are also shown separated by fatty acid saturation state. All graphs show lipid levels of *Sms1*^-/-^ testes (red) in comparison to *Sms1*^*WT*^ testes (green), which were set to 100%. Data is presented as mean percentage ± SEM (*Sms1*^*WT*^, n = 15; *Sms1*^-/-^, n = 17); *p≤0.05, **p≤0.01, ***p≤0.001.

Next, to determine SMS1 enzyme activity *in vivo*, we established lipid profiles from testes of *Sms1*^*+/+*^ vs. *Sms1*^-/-^ littermates. Substrates of SMS1, Cer and PCs such as diacylphosphatidylcholines (PCaa) and phosphatidylcholine-plasmogenes (PCae) as well as SM and lyso-phosphatidylcholines (lysoPC) of different chain length and saturation states were analysed. In comparison to wild-type levels, SMs were found reduced to 78.9 ± 2.0% (p<0.0001) in *Sms1*^-/-^ animals compared to wild-types (Fig 2A). Overall Cer levels appeared almost unaffected in testis samples (Fig 2A), which was not surprising considering the role of SMS2 and the multiple effects of Cer as cytostatic, inflammatory and pro-apoptotic factor and the function of Cer as a crucial branching point for several other lipid pathways [32]. A detailed analyses of Cer species however revealed shifts in their relative amounts in testis. Namely, there was a significant increase in the C18:1/18:1 species relative to most other Cer species, with the exception of C18:1/26:1 (S3 Table).

Analysis of PCaa revealed a reduction to 91.9 ± 2.4% (p = 0.0089) in *Sms1*^-/-^. Detailed analysis pointed out, that this unexpected reduction was due to low levels of unsaturated PCaa (91.3 ± 2.4%; p = 0.0056), while the amount of saturated PCaa was comparable to wild-type levels (Fig 2C). Neither PCae levels of total saturated, nor of total unsaturated species were altered in *Sms1*^-/-^ samples, compared to wild-type levels. (Fig 2C).

A detailed analysis of the most abundant PCaa and PCae species of *Sms1*^-/-^ against wild-type levels revealed a significant reduction in mono- (MUFA) and PUFA containing species (Table 1A).

**Table 1:** Lipid profile of *Sms1* mutant testes PCaa, PCae and lysoPC species, compared to *Sms1* wild-type levels. Lipid levels with the highest intensities are shown for diacylphosphatidylcholines (PCaa, A) plasmalogens (PCaes, B) and lyos-phosphatidylcholines (lysoPCs, C). Additionally, predicted major fatty acids are listed. Data is presented as mean percentage ± SEM, with wild-type levels set to 100% (*Sms1* wild-type, n = 15; *Sms1* mutant, n = 17); *p≤0.05, **p≤0.01, ***p≤0.001.

**A**									
**PC aa species**	**Sms1WT (n = 15)**			**Sms1MUT (n = 17)**			**significance**		
	**Intensity**	**%**	**SEM %**	**Intensity**	**%**	**SEM %**	**ttest**	**significance**	
PC aa total	10.445.747,2	100,0	1,6	9.602.724,8	91,9	2,4	0,0089	**	
PC aa saturated	560.581,0	100,0	1,5	573.084,7	102,2	2,5	0,4548		
PC aa unsaturated	9.885.166,2	100,0	1,6	9.029.640,1	91,3	2,4	0,0056	**	
PC aa CX:1	2.430.350,1	100,0	1,5	2.400.435,1	98,8	2,2	0,6527		
PC aa CX:2	724.363,8	100,0	2,1	637.657,7	88,0	2,7	0,0016	**	
PC aa CX:3	829.606,0	100,0	1,8	729.132,2	87,9	2,6	0,0008	***	
PC aa CX:4	2.744.306,4	100,0	1,7	2.572.597,0	93,7	2,3	0,0353	*	
PC aa CX:5	2.326.950,0	100,0	1,7	1.977.047,8	85,0	2,4	0,0000	***	
PC aa CX:6	829.590,0	100,0	2,3	712.770,3	85,9	3,2	0,0015	**	predicted major FA*
PC aa C38:5	1.950.613,7	100,0	1,6	1.641.726,7	84,2	2,4	0,0000	***	22:5 20:5 20:4 18:0 18:1 16:0
PC aa C34:1	1.798.062,7	100,0	1,3	1.786.400,0	99,4	2,1	0,7968		
PC aa C36:4	1.618.574,5	100,0	1,4	1.506.246,7	93,1	2,4	0,0210	*	20:4 18:2 16:0
PC aa C38:4	981.770,8	100,0	2,3	928.077,1	94,5	2,3	0,1033		
PC aa C38:6	585.682,2	100,0	2,2	486.001,3	83,0	3,6	0,0006	***	22:6 22:5 20:5 20:4 18:1 18:2 16:0 16:1
PC aa C36:3	561.250,4	100,0	1,8	470.413,3	83,8	3,0	0,0001	***	18:1 18:2
PC aa C36:1	456.552,7	100,0	2,9	458.580,7	100,4	3,4	0,9221		
PC aa C32:0	380.136,1	100,0	1,4	407.911,6	107,3	2,7	0,0259	*	16:0
PC aa C34:2	357.430,0	100,0	2,5	284.355,8	79,6	3,0	0,0000	***	18:1 18:2 16:0 16:1
**B**									
**PC ae species**	**Sms1WT (n = 15)**			**Sms1MUT (n = 17)**			**significance**		
	**Intensity**	**%**	**SEM %**	**Intensity**	**%**	**SEM %**	**ttest**	**significance**	
total PC ae	2.308.270,3	100,0	2,015916125	2.276.965,1	98,6	2,957074382	0,707889085		
saturated PC ae	135.666,1	100,0	1,8	133.692,1	98,5	2,8	0,6667		
unsaturated PC ae	2.172.604,2	100,0	2,0	2.143.273,0	98,6	3,0	0,7113		
PC ae CX:1	445.537,8	100,0	2,3	526.085,6	118,1	4,3	0,0012	**	
PC ae CX:2	152.594,7	100,0	2,3	152.839,3	100,2	2,9	0,9659		
PC ae CX:3	108.639,9	100,0	2,2	110.662,7	101,9	2,9	0,6097		
PC ae CX:4	390.514,1	100,0	2,3	393.153,3	100,7	3,3	0,8680		
PC ae CX:5	595.251,5	100,0	2,2	554.955,0	93,2	3,0	0,0751		
PC ae CX:6	480.066,2	100,0	2,2	405.577,0	84,5	3,2	0,0005	***	predicted major FA*
PC ae C38:5	440.374,7	100,0	2,2	408.857,6	92,8	3,1	0,0682		
PC ae C38:6	435.848,4	100,0	2,2	361.283,8	82,9	3,3	0,0002	***	22:6 22:5 20:5 20:4 18:1 18:2 16:0 16:1
PC ae C34:1	283.880,8	100,0	2,9	373.994,7	131,7	5,6	0,0001	***	18:0 18:1 16:0 16:1
PC ae C36:4	207.666,1	100,0	2,5	204.586,7	98,5	3,6	0,7362		
PC ae C38:4	121.854,9	100,0	2,2	123.640,4	101,5	3,1	0,7023		
PC ae C40:5	92.162,4	100,0	2,5	92.796,8	100,7	3,3	0,8679		
PC ae C32:1	77.985,9	100,0	1,7	66.306,6	85,0	2,6	0,0001	***	18:0 18:1 16:1 14:0 14:1
PC ae C34:2	73.001,5	100,0	3,2	67.181,1	92,0	4,0	0,1318		
PC ae C40:0	55.709,2	100,0	2,6	54.824,8	98,4	3,2	0,7015		
PC ae C36:5	51.057,4	100,0	2,2	42.015,7	82,3	2,5	0,0000	***	20:5 20:4 16:0 16:1
**C**									
**lyso PC a species**	**Sms1WT (n = 15)**			**Sms1MUT (n = 17)**			**significance**		
	**Intensity**	**%**	**SEM %**	**Intensity**	**%**	**SEM %**	**ttest**	**significance**	
total lyso PC a	434.258,4	100,0	5,9	294.251,0	67,8	5,2	0,0003	***	
Lyso PC 16:0	160.184,4	100,0	6,2	102.757,1	64,1	6,0	0,0002	***	
Lyso PC 20:4	91.537,0	100,0	7,6	58.978,2	64,4	6,1	0,0010	***	
Lyso PC 18:1	71.646,4	100,0	6,9	50.157,9	70,0	5,9	0,0025	**	
Lyso PC 18:0	58.547,4	100,0	4,7	43.893,5	75,0	4,6	0,0007	***	
Lyso PC 20:3	17.463,1	100,0	7,7	10.297,6	59,0	6,1	0,0003	***	
Lyso PC 18:2	15.400,3	100,0	7,9	8.920,0	57,9	5,1	0,0001	***	
Lyso PC 14:0	3.701,9	100,0	1,7	3.340,3	90,2	1,3	0,0001	***	
Lyso PC 16:1	3.526,8	100,0	6,3	2.312,6	65,6	5,2	0,0002	***	
Lyso PC 26:0	3.344,8	100,0	10,3	4.216,3	126,1	19,2	0,2444		

Among the FAs with a highly significant reduction, we found the most striking reduction for the PCaa species C38:5 and C38:6, indicative of a loss of arachidonic acid (AA; C20:4), EPA (C20:5), docosapentaenoic acid (DPA; C22:5) or DHA (C22:6) all of which belong either to n-3 or n-6 FAs (Table 1A). While total PCae were unaltered, again PCae C38:6 was among the strongly reduced lipids (Table 1B).The total level of lysoPCs was significantly reduced to 73.4 ± 0.2% (p = 0.001) in testes of *Sms1*^-/-^ males in comparison to the level in wild-type samples (Fig 2B). This reduction was present in all major lysoPC species (Table 1C). These results suggests that SMS1 has an unexpected role in testis PC, including n-3/6 PUFA, homeostasis.

### *Sms1*^-/-^ males show display altered luminal contents of testes and epididymides

**Fig 3:**
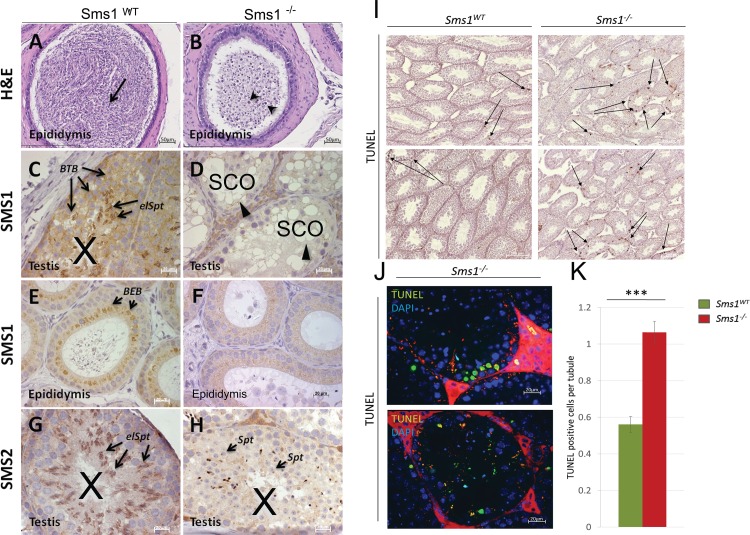
Histological analysis of testes and epididymides of *Sms1*^-/-^ males indicates impaired spermatogenesis. (A, B) H&E staining of *Sms1*^*WT*^ (A) and *Sms1*^-/-^ (B) accumulation of round cells (black arrow heads) in the *Sms1*^-/-^ epididymal lumen, instead of Sz (black arrow in A). Scale bar: 50μm. (C–F) IHC for SMS1. (C,E) *Sms1*^*WT*^; (D,F) *Sms1*^-/-^. (G, H) IHC for SMS2. (G) *Sms1*^*WT*^, (H) *Sms1*^-/-^. Scale bar: 20μm. (I, J) TUNEL staining of *Sms1*^*WT*^ and *Sms1*^-/-^ testes. (I) Apoptosis (black arrows) in *Sms1*^*WT*^ seminiferous tubules (left panel), *Sms1*^-/-^ (right panel, scale bar: 200μm). (J) Higher magnification revealed apoptotic clusters near the basal lamina and apoptotic material in the tubule lumen of *Sms1*^-/-^ seminiferous tubules. (K) Quantification of TUNEL-positive events per tubule revealed a higher rate of apoptosis in *Sms1*^-/-^ testes compared to *Sms1*^*WT*^. Scale bar: 20μm. Blood-testis-barrier (BTB), blood-epididymis barrier (BEB), spermatocyte (Sc), Sertoli-cell only phenotype (SCO), spermatozoa (Sz), spermatid (Spt), elongating spermatid (elSpt).

**Fig 4:**
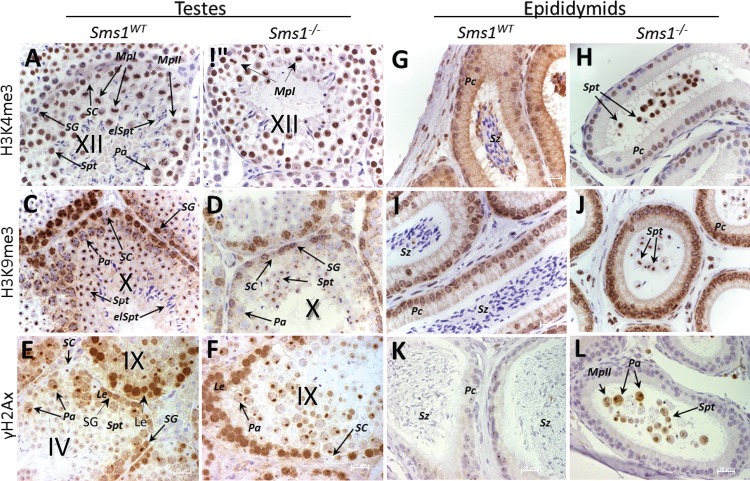
Specification of stage specific histone markers during spermatogenesis on testes and epididymides sections of *Sms1*^-/-^ compared to *Sms1*^*WT*^ animals illustrates sloughing of immature spermatogenesis stages. (A-L) IHC of specific histone variants on sections of *Sms1*^*WT*^ and *Sms1*^-/-^. (A-F) stage-matched seminiferous tubules and (G-L) epididymides. (A, G) IHC of H3K4me3 in *Sms1*^*WT*^ with weak staining of SC and SG nuclei. (B, H) H3K4me3 in *Sms1*^*-/-*^. (C, I) H3K9me3 in *Sms1*^*WT*^ was strongest in SG and Pa and was also found in Spt. (I) In epididymides H3K9me3 was mainly found in Pc. (D, J) H3K9me3 in *Sms1*^-/-^. (E, K) γH2AX in *Sms1*^*WT*^, marked B-type SG and XY-bodies in Pa and early Spt stages. (F, L) γH2AX expression in *Sms1*^-/-^. (L) γH2AX staining of *Sms1*^-/-^ epididymides detected sloughed Pa, MpII stages and Spt in the epididymal lumen. Tri-methylated lysine 4 of histone 3 (H3K4me3); tri-methylated lysine 9 of histone 3 (H3K9me3); elongating spermatids (elSpt) leptotene spermatocytes (Le), pachytene spermatocytes (Pa), Metaphase I (MpI) Metaphase II (MpII), Principal cells (Pc); Sertoli cell (SC), spermatogonia (SG), spermatozoa (Sz), spermatids (Spt). The stages of the seminiferous tubules are indicated by roman numbers. Scale bar: 20μm.

In epididymides of *Sms1*^-/-^ males, mature spermatozoa were rarely present and instead, round spermatids, lacking acrosome structures and tails, were accumulated (Fig 3A and 3B). Sms1 expression indicates two expression domains in seminiferous tubules: 1. in Sertoli cells forming the blood-testis-barrier (BTB) and 2. in elongating spermatid cells (Fig 3C and 3D). In epididymis, Sms1 expression was detected in principal cells, which are forming the blood-epididymis-barrier (BEB; Fig 3E and 3F). In *Sms1*^-/-^ seminiferous tubules of different stages, large vacuoles were frequently encountered (Fig 3D; see also Fig 4B and 4F). The Sms1 expression in testis and epididymis was also reflected by the β-gal reporter (see S2A and S2B Fig). In contrast, SMS2 protein expression was found expressed along with SMS1 in elongating spermatids and spermatozoa, where both co-localized to the developing acrosome [33]. This expression domain was strongly reduced in *Sms1*^*-/-*^ males (Fig 3G and 3H). Compared to the wild-type, round and elongated spermatids were strongly reduced in number and only occasional spermatozoa were observed in testis tubules of *Sms1*^-/-^ males (Fig 3G and 3H).

Furthermore, apoptotic cells were noted in seminiferous tubules of *Sms1*^-/-^ testes (Fig 3I and 3J) and wild type (not shown). Furthermore, often there was accumulation of apoptotic material in the lumen of the tubules (Fig 3J). Quantification of TUNEL-positive cells per tubule revealed a 1.9-fold higher rate of apoptosis in *Sms1*^-/-^ testes (1.06 ± 0.06 TUNEL-pos. cells/tubule) compared to wild-types (0.56 ± 0.04 TUNEL-pos. cells/tuble; Fig 3K). These observations were made using animals of 20 weeks of age and in comparison to *Sms1*^*+/+*^ males. Further investigation of the number of apoptotic cells in TUNEL-positive tubules of stage XII and I-XI revealed, however, that the number of dying cells/tubule was similar in mutant and wild-type testes. In average there were 3.43 apoptotic cells per stage XII wild-type tubule (+-0.40 SE) versus 3.71 (+-0.47) cells/tubule in the mutant. Cell death-positive stage I-XI tubuli (in the absence of haploid cells staging could only be crudely done) displayed in average 3.68 (+-0.29) and 4.0 (+-0.28) apoptotic cells per wild-type and mutant tubule, respectively. Our data show that there is an overall increase of cell death in the mutant, which likely relates to the disruption of the BTB and the spermiogenic epithelium. Similar rates of apoptosis in stage XII tubules furthermore indicate that spindle defects or chromosomal missegregation are not increased in the mutant.

### Sloughing involves spermiogenic cells

From our observations, we hypothesized sloughing of mainly elongating spermatid stages into testis lumen of the mutants and their accumulation in the epididymides. In order to determine, the exact developmental stage at which the germ cells of *Sms1*^-/-^ males lose their contact to Sertoli cells, we analysed the expression of characteristic histone modifications. Histone modifications during meiosis include trimethylation of histone H3 at lysine residue 4 (H3K4me3) prior to meiotic foci formation, a modification that is associated with active chromatin [34–36]. We determined the expression of H3K4me3 and found this histone modification to be expressed at normal intensity from the spermatogonia stage to the end of meiosis II in both, wild-type (Fig 4A) and *Sms1*^-/-^ testes (Fig 4B). In both genotypes, H3K4me3 was weakly expressed in Sertoli cells but detectable in pachytene spermatocytes (Fig 4A and 4B).

Histone H3 lysine 9 becomes first trimethylated (H3K9me3) in spermatogonia, a modification that is associated with inactive chromatin. While H3K9me3 is not found in the XY-body of pachytene spermatocytes, it is detectable in the heterochromatin clusters of spermatogonia until the round spermatid stages [37,38]. This modification was seen in wild-type as well as in testes of *Sms1*^-/-^ animals (Fig 4C and 4D). Wild-type epididymal spermatozoa have already condensed their chromatin and thus, wild-type epididymal spermatozoa were negative for both H3K9 and H3K4 trimethylation (Fig 4G and 4I). In the epididymides of *Sms1*^*-/-*^ animals, trimethylation of both H3K9 and H3K4 was determined in spermatocytes and spermatids, indicating sloughing of histone-retaining spermatogenic cells up to spermatid stages (Fig 4H and 4J).

Histone H2AX becomes phosphorylated at serine residue 139 (γH2AX) [39] during S-phase and meiotic prophase [40,41]. γH2AX formation occurs during S-phase in B-spermatogonia and remains detectable until the pachytene-stage, where it localizes to the XY-body [42]. This histone modification is only faintly detectable in spermatid heterochromatin while it occurs during chromatin remodeling in spermatids of step 8–10 (Fig 4E and 4F; [34,35,37]). γH2AX-positive cells were not observed in the epididymal lumen of wild-type mice (Fig 4K), while γH2AX-positive spermatocytes and haploid spermatids were present in mutant epididymides (Fig 4L), indicating that sloughing involved pachytene to late spermatid stages.

### The blood-testis barrier appears compromised in *Sms1* mutants

**Fig 5:**
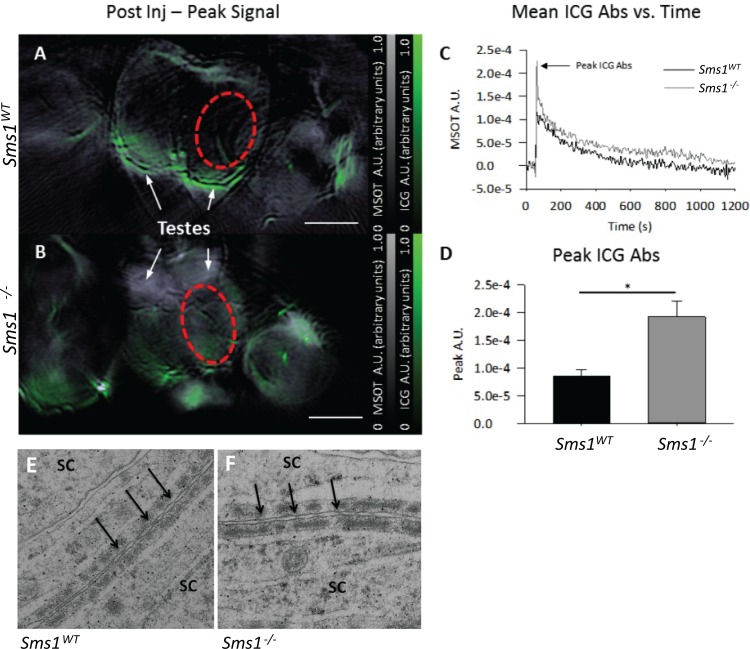
blood testes barrier appeared to be established but was compromised in *Sms1*^-/-^. (A, B) Unmixed ICG signal (green) overlayed on anatomical images (reconstructed image at 860nm) of *Sms1*^*WT*^ and *Sms1*^-/-^ animals immediately following ICG injection. White horizontal scale bars correspond to 3 mm and colormaps are shown for the overall absorbance signal in the anatomical images and (grey) and multispectrally-unmixed ICG signal (green). Mean pixel intensity was determined in the area of the testes, as indicated by the red ellipsoids in panels A and B, and plotted vs. time. (C) Representative ICG-absorbance vs. time curves for both *Sms1*^*WT*^ (black trace) and *Sms1*^-/-^ (grey trace) animals. MSOT A.U. values were normalized by subtracting the overall signal by the baseline signal measured prior to ICG injection. (D) Peak values of the ICG absorbance vs. time curves. A.U., absorption units. Values are shown as mean ± SEM; *Sms1*^*WT*^, n_obs_ = 3 from 3 animals; *Sms1*^-/-^, n_obs_ = 6 from 3 animals; *, p < 0.05. (E) Transmission electron microscopy (TEM) of Sertoli cell (SC) boundaries in the testes tubules of *Sms1*^*WT*^ and *Sms1*^-/-^ mice. Tight-junctions (black arrows), necessary for the formation and integrity of the BTB between SC were detectable. Scale bar: 20μm; TEM x 50000.

We hypothesized that the specific reduction of PUFAs in *Sms1*^-/-^ testes may cause sloughing of spermatids due to a disturbance of the integrity of the BTB, as proposed by Stoffel et al. [43]. The BTB, built by special tight-, gap- and adherens-junction proteins between Sertoli cells, separates the adluminal stages of spermatogenesis from the blood supply and the cellular immune system, thereby creating and controlling a specific microenvironment required for sperm maturation (for a recent review see: [44]. In order to test the hypothesis of a compromised BTB, we quantified the expression of representative junction markers in testical and epididymal protein samples by western blotting (S1G Fig). Interestingly β-catenin (283.3 ± 26.0%, p = 0.0054) and connexin 43 (112.0 ± 1.7%, p = 0.0084) as subunits of adherens- or gap-junctions, respectively, were significantly elevated in testes of *Sms1*^-/-^ males (S1G and S1H Fig), while their protein levels in the epididymides were unchanged. Occludin levels in the mutants showed a tendency to be elevated in testes (149.1 ± 18.7%, p = 0.0776) but were unaltered in the epididymides compared to wild-type levels (Fig 1G and 1H). Immunostaining, using anti-β-catenin and anti-connexin 43 antibodies did not reveal any dislocation of junctional proteins (S1A, S1B, S1D, and S1E Fig), while occludin was more abundantly expressed in *Sms1*^-/-^ (S1C and S1F Fig). Further studies of BTB integrity were done by transmission electron microscopy (TEM). It appeared that tight junctions between Sertoli cells were established equally in wild-type and *Sms1*^-/-^ testes (Fig 5E and 5F). In order to elucidate the possibility of a functionally compromised BTB *in vivo*, Multi-Spectral Optoacoustic Tomography (MSOT) imaging of Indocyanine Green (ICG) distribution in the testes was performed. Following ICG injection, wild-type testes show little ICG peak signal within testes, while, a qualitatively higher signal was detected in *Sms1*^-/-^ testes (Fig 5A and 5B). Representative time courses of ICG distribution in the *Sms1*^-/-^ and wild-type mice are shown in Fig 5C, indicating higher peak ICG levels in the *Sms1*^-/-^ animals (19.21e^-5^ ± 2.88e^-5^ optical absorbance units (A.U.) when compared to the wild-type (8.43e^-5^ ± 1.25e^-5^ A.U., p = 0.040, Fig 5D). The clearance of ICG from the testes was also delayed in the *Sms1*^-/-^ mice when compared to wild-type controls. However, this trend was not significant. The higher ICG signal in the *Sms1*^-/-^ animals indicated increased permeability of the BTB, since such loss of integrity would result in ICG leaking from vessels to testicular tissue.

### *Sms1*^-/-^ animals show subfertility or infertility, which is age-related

In agreement with a previous report [45] we noted that *Sms1*^-/-^ males had reduced reproductive capabilities. To systematically analyze the fertility of *Sms1* males at two different ages, twelve male animals covering all genotypes were analysed (*SMS1*^*+/+*^ n = 2, *Sms1*^+/-^ n = 4, *Sms1*^-/-^ n = 6). We first determined the fertility status at the age of eight weeks. *Sms1* males were mated to C57Bl/6J females (age: 11 weeks) for a three week period. All wild-type and *Sms1*^+/-^animals were able to produce offspring, four of the six *Sms1*^-/-^ animals were fertile (66%), while two failed to produce offspring with eight to eleven weeks of age (33%).

**Fig 6:**
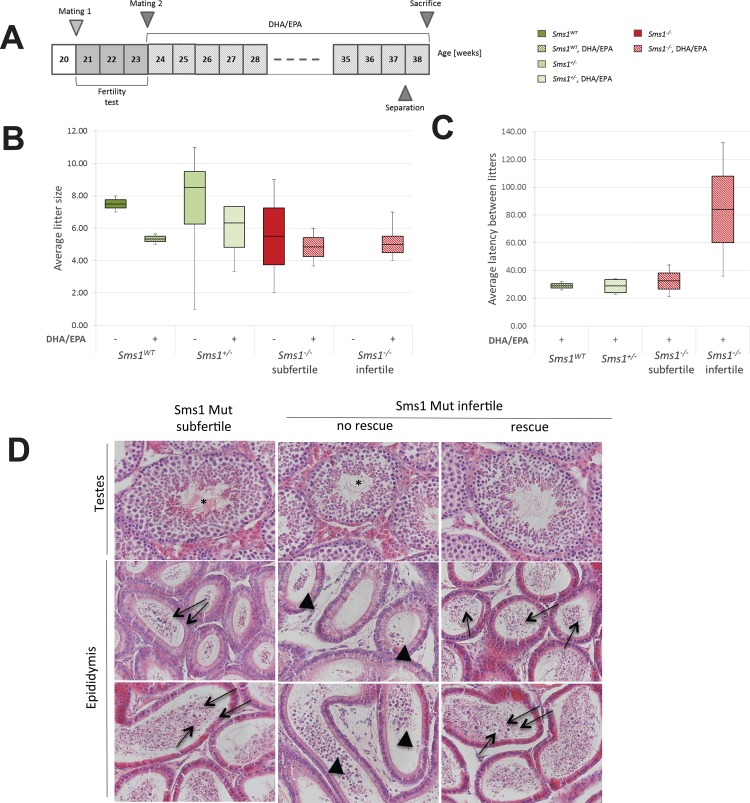
PUFA enriched diet ameliorates the sloughing phenotype of infertile *Sms1*^-/-^. (A) Schematic time course of the infertility test mating scheme, with subsequent PUFA supplementation. *Sms1*^*WT*^ (n = 2), *Sms1*^+/-^ (n = 4) and *Sms1*^-/-^ (n = 6) were mated at 20 weeks for a fertility test (mating 1). After three weeks females were replaced by fresh females (mating 2). Mating 2 was maintained for 13 weeks and PUFA supplementation started with mating 2. At 38 weeks testes were analysed. (B) Mean number of offspring produced by *Sms1*^*WT*^ (dark green boxes), *Sms1*^+/-^ (light green boxes), *Sms1*^-/-^ subfertile and *Sms1*^-/-^ infertile (red boxes) without (filled boxes) and with FA diet supplementation (dashed boxes). (C) Mean latency between litters with PUFA supplementation. (D) Representative H&E staining of testes sections after 14 weeks of PUFA supplementation. Images show sections from testes and epididymides from subfertile, infertile not rescued and infertile rescued *Sms1*^-/-^ after the diet. Arrows indicate round spermatids. Black arrow heads indicate accumulation of immature germ cells in infertile not rescued *Sms1*^-/-^. Asterisks in subfertile and rescued infertile *Sms1*^-/-^ indicate apparently normal spermatogenesis. Scale bar: 50μm. Data is shown as median ± quartiles.

At the age of 20 weeks we observed the described testis phenotype in six out of six mutant males (compare Fig 3A and 3B), which also impacted the fertility state of *Sms1* males. While all wild-type and *Sms1*^+/-^ males tested produced offspring, only two out of the six male *Sms1*^-/-^ appeared fertile (33%), whereas four failed to produce any offspring (66%). These results pointed to an early age-related decrease of total fertility in *Sms1*^-/-^ males compared to *Sms1*^+/-^and wild-type animals, starting already at the age of 8 weeks. We also analysed the litter size. Infertility was assumed in this experiment when a male failed to produce offspring during the mating time of 3 weeks (Fig 6A). Fertile *Sms1*^-/-^ males produced 5.50 (IQR: 3.50) pups per litter, while *Sms1*^+/-^ and wild-type males produced 8.50 (IQR: 3.25) and 7.50 (IQR: 0.50) pups per litter, respectively (Fig 6B). At the age of 20–23 weeks the fertile *Sms1*^-/-^ males were considered subfertile as compared to wild-type and *Sms1*^+/-^ males.

### Ameliorated spermatogenesis phenotype in *Sms1*^-/-^ males treated with a PUFA diet

The observed reduction in AA, EPA, DPA or DHA (Tab1A, B) and the profound role of PUFAs in spermatogenesis suggested an involvement for the observed male sub-/infertility phenotype (Fig 6B). As AA treatment was expected to be little suitable as a potential therapeutic intervention we supplemented males with a diet of 38% DHA and 46% EPA, in order to investigate whether the subfertility/infertility could be reversed.

The dietary supplementation experiment was initiated following fertility analysis. Once, the fertility status of the males was determined, *Sms1* males of all genotypes were remated to eleven week old C57Bl/6J females and were manually fed an oral dose of the n-3 FA containing oil on a daily basis for 13 weeks as described in Materials and Methods (Fig 6A). During the testing period *Sms1*^*+/+*^ and subfertile *Sms1*^-/-^ males produced an average of 2.50 ± 0.50 litters, each. *Sms1*^+/-^ males produced 3.00 ± 0.00 litters. Of the confirmed infertile *Sms1*^-/-^ males 50% produced offspring with feeding, while the other 50% failed to generate offspring. *Sms1*^-/-^ animals with rescued fertility produced one litter each during the testing period of 13 weeks. We noted, that for *Sms1*^*+/+*^ and *Sms1*^+/-^ males the number of offspring was reduced with the feeding of the n-3 FA diet to an average of 5.33 (IQR: 0.33) pups per litter for *Sms1*^*+/+*^ and to 6.33 (IQR: 2.50) pups per litter for *Sms1*^+/-^ males (Fig 6B). This was likely due to the stress that was associated with the daily manual feeding procedure. Previously subfertile males also produced less offspring after feeding (4.84 (IQR: 1.17) pups per litter). Previously infertile males, produced an average of 5.00 (IQR: 1.00) pups per litter (Fig 6B). We determined the average latencies between litters to check for prolonged reproduction times. The results for *Sms*^*+/+*^ (24.00 (IQR: 1.0) days) and *Sms1*^+/-^ males (23.00 (IQR: 2.5) days) were similar (Fig 6C). Subfertile *Sms1*^-/-^ and infertile *Sms1*^-/-^ males required more time for reproduction (subfertile *Sms1*^-/-^: 31.50 (IQR: 9.5) days, infertile *Sms1*^-/-^: 84.00 (IQR: 48.0) days; Fig 6C). Natural occurrence of unproductive matings in the strain C57BL6/6J is 12.6% (http://phenome.jax.org/db/qp?rtn=views/measplot&brieflook=14934&ustrainids=7&brieflooktool=&projhint=Jax3), which insufficient to explain the amount of infertility observed in these matings.

All offspring was heterozygous, confirming homozygosity of the males, which was also confirmed by re-genotyping via triplex-PCR. Matings were terminated and males sacrificed after one additional week of PUFA enriched diet. As shown in Fig 6D, spermatogenesis had progressed almost normally in subfertile *Sms1*^-/-^ males after 14 weeks of PUFA diet. Only few round spermatids were visible in mutant epididymides while the majority of cells had matured into spermatozoa (Fig 6D). In contrast infertile *Sms1*^-/-^ males, which could not be rescued still showed impaired spermatogenesis and accumulation of immature germ cells in the epididymal cauda (Fig 6D). In previously infertile *Sms1*^-/-^ animals, which produced offspring after PUFA diet, the histology of seminiferous tubules ranged from being ‘impaired’ up to ‘fully restored’. Their epididymal tubules were similar to those of subfertile *Sms1*^-/-^ males with a few round, but a majority of elongated germ cells (Fig 6D).

## Discussion

In sperm cell membranes, phospholipids represent the most prominent lipid fraction with a high content of PUFAs, e.g. VLC-PUFA-SM, which have a role in sperm capacitation, but are also found in Sertoli cells [46–48]. SM is generated by two different SMSs–SMS1 and SMS2. Redundant and non-redundant functions of both SMSs in different organs were illustrated by knockout animals of *Sms1* [17,49,50], and *Sms2* [51,52]. While no spermatogenesis or fertility defects and normal reproduction were reported from *Sms2* knockout animals [51,53,54] impaired reproduction has been noted from male *Sms1* knockout animals [45]. Unlike Dong et al., [45] where complete infertility of male *Sms1* knockout animals was seen, we observed residual age-dependent fertility in a number of mice, which might be explained by different genetic background or potential differences in diet and age of the animals analysed. In the present study, we show, that disruption of *Sms1* expression leads to distinct changes in the testicular lipid profile, especially concerning unsaturated FA containing species and sloughing of pachytene to late spermatid stages. The testicular SM levels of *Sms1*^-/-^ males were 20% reduced as compared to wild-type. This reduction is in the expected range [17,45] with the remaining 80% likely resulting from SMS2 activity and dietary uptake. One explanation for the different impact of SMS1 and SMS2 on male fertility is the subcellular localization and functional differences of both enzymes. SMS1 activity is restricted to the Golgi apparatus, while SMS2 mainly locates to the plasma membrane where it is important for Cer homeostasis [10,55] SMS1 has a role in the maintenance of intracellular SM homeostasis, normally generating the bulk SM pool, which also supplies the plasma membrane [7]. The imbalance in the lipid metabolism, caused by disruption of *Sms1* therefore affects intracellular, but also cell surface lipid composition, altering protein delivery, enzymatic activity and lipid based cell signaling.

### The Sms1 mutation altered the lipid profile of mutant testis

Although Cer and PC are the direct substrates of SMS1, their overall levels did not rise in *Sms1*^-/-^ testes, rather *Sms1*^-/-^ maintained steady levels of otherwise potentially cytotoxic Cer. This is in agreement with Cer homeostasis to be maintained mainly by enzymes other than SMS1, which was also seen for *Sms1* knockout mice, which had only marginal effects on Cer levels, while a *Sms2* depletion led to Cer elevation in plasma [17,52,54]. Parallel to unaltered total Cer, unsaturated PCaa and PCae species were decreased. In the PCaa and less pronounced in the PCae profile, we found a decrease of specific PUFA (AA, EPA, DPA and DHA) containing lipids and an overall reduction in MUFA/SFA and PUFA/SFA ratios, which likely reduced the ability of Sertoli cells to support accumulation of LC-PUFA and VLC-PUFA in spermatozoa [56]. The production of these PUFA-SMs appears to be primarily derived from the activity of SMS1. The observed reduction in several unsaturated testicular PCaes, one of the major phospholipids of sperm membranes, together with an alteration in SM levels, leads to changes in membrane properties and thus affects the special composition needed for interaction and communication between Sertoli cells and spermatocytes necessary for the differentiation into mature sperm.

### BTB functionality was compromised in Sms1^-/-^ males

As described, immature spermatogenic cell types ranging from pachytene to late spermatid stages were sloughed and accumulated in the epididymal lumen of *Sms1*^-/-^ males. Several lines of evidence support a contribution of BTB malfunctioning to the phenotype. Usually, the BTB is crossed by spermatocytes between preleptotene and leptotene stage, while meiotic prophase stage cells are held back, for as long as they are primed for the passage and provided with their special set of proteins and lipids. The BTB is made up of several distinct junction types, which set up a dynamic network between Sertoli/Sertoli and Sertoli/germ cells, regulating the passage of germ cells from the basal to the adluminal compartment [44,57]. Indications for a BTB deficiency in *Sms1*^-/-^ males arose from altered junction protein levels, such as elevated β-catenin and connexin-43, which represent subunits of adherens- and gap-junctions, respectively [44,56]. Connexins are continuously synthesized and degraded representing a highly flexible system for intercellular contact and communication, known to influence expression levels of other junctional proteins [44,58,59]. An increase in junction proteins may result from disrupted Trans-Golgi-network (TGN)-mediated protein trafficking and protein secretion, which was shown to be dependent on SMS activity [60]. Although the subcellular localization of junctional proteins was found mostly unaltered in *Sms1*^-/-^ males, their elevated expression indicates an impaired Sertoli cell/germ cell interaction. The protein levels of occludin, one of the major component of tight-junctions, tended to be elevated as well in *Sms1*^-/-^ males. Our analysis of the structure of tight-junctions between Sertoli cells by TEM did not reveal obvious morphological differences in the *Sms1*^-/-^ in comparison to wild-type testes, however, the BTB is not established through tight-junctions only. Adherens-junctions, gap-junctions, channel proteins and transporters also play important roles in intercellular contacts and communication [61]. After injection of ICG into tail veins of living animals followed by life imaging using MSOT we determined a significantly higher fluorescent signal from the testes lumen of *Sms1*^-/-^ animals, clearly indicating BTB dysfunction and leakiness.

### PUFA diet ameliorated the sloughing phenotype of infertile Sms1^-/-^

In breeding experiments the majority of *Sms1*^-/-^ males were either infertile or subfertile. We noted that the grade of infertility was age-related, as indicated by a progressive loss in number of offspring and a diminished ability to reproduce.

A crucial role for PUFAs for male fertility has been proposed by a number of studies in different organisms [62–65]. Epidemiological studies suggested a negative impact of a diet rich in saturated fatty acids ("Western diet") on the sperm quality in young men [66]. Evidence for the importance of PUFAs for the integrity of the BTB has been recently suggested, after analyzing Δ^6^-desaturase (*Fads2*) mutant mice [43,67]. The reduction in PUFA levels, phenotypic similarities of *Sms1* and *Fads2* mutants in respect to spermatogenesis, including a significant decrease in PUFA-PCaa and possible leakiness of the BTB prompted us to supplement the diet of *Sms1*^-/-^ with n-3 PUFAs (DHA/EPA). PUFA supplementation reconstituted the fertility status in 50% of these animals. By taking into account that pregnancies last approximately three weeks and that the first litter was obtained 36 days after mating, we calculated in retrospect that treatment for a minimum of approximately two weeks or more with a PUFA supplementation was needed to restore fertility in *Sms1*^-/-^ males. Given that a full cycle of spermatogenesis takes 34 days in mice and is made up of four subcycles (8.6 days each), our data support the hypothesis that PUFA supplementation affects pachytene spermatocytes and spermatid stages [68]. Rabionet et al. [69], suggested that SM (C16) and glycosphingolipid production in mouse testis starts around prophase and that after passing the BTB, with the onset of *CerS3* expression, the cells switch to the production of VLC-PUFAs, which would be in good agreement with our findings, although we can not exclude other mechanisms to be involved. More recently, Rabionet and coworkers demonstrated through a germ cell-specific knockout of *CerS3* that the enzyme is crucial for the formation of intercellular bridges (ICB) between syncytial spermatids. ICB stability is based on ultra long polyunsaturated SMs. Similarly to *Sms1*^-/-^ animals, the germ cell-specific *CerS3* knockout resulted in elevated apoptosis of spermatogenic cells [13]. The sloughing phenotype of *Sms1*^-/-^ was significantly ameliorated after dietary supplementation. The histological analysis revealed high numbers of mature spermatozoa in epididymides and only few round spermatids of PUFA-fed *Sms1*^-/-^ males, which produced offspring. This suggests further that a longer treatment duration might help to restore male fertility further.

In summary, the exact cause for sloughing of germ cells in the *Sms1*^-/-^ males is not entirely clear, but our findings indicate the following mechanisms to be involved. 1) A dysfunctional BTB, which is initiated in *Sms1*^-/-^ males, as was demonstrated by the presence of tight junctions by TEM and marker analysis, but also appeared leaky as was shown by MSOT. 2) A hampered switch to the production of LC-PUFAs, which appears to gain importance after the passage of the BTB, as it was shown by the reduction in AA, EPA, DPA and DHA containing PCaas and PCaes 3) Abnormal regulation of intercellular contacts and communication after the passage of the BTB, indicated by the increase of adluminal junctional proteins.

## Supporting Information

S1 Fig Analysis of junctional markers reveals testes-specific elevation. **(**A-F) Immunohistochemistry (IHC) for junctional marker proteins. (A, B, C) *Sms1*^*WT*^; (D, E, F) *Sms1*^-/-^ animals (A, D) β-catenin (β-CAT) at the site of the BTB (black arrows). (B, E) Connexin-43 (CX-43). (C, F) Occludin. (G) Immunoblot for β-catenin, connexin-43 and occludin from testes and epididymides of *Sms1*^*WT*^ and *Sms1*^-/-^ males (n = 3 per genotype). (H) Quantification of β-catenin, connexin-43 and occludin testes protein levels. *Sms1*^*WT*^ protein levels were set to 100%. Data is presented as mean percentage ± SEM (n = 3 per genotype); ^ns^p≥0.05,*p≤0.05, **p≤0.01, ***p≤0.001.
(TIFF)

S2 Fig β-gal expression in testis and epididymis in *Sms1*^*+/-*^ males. A) β-gal activity in testes of heterozygous males reflects wild-type expression of Sms1. The staining pattern indicates a dual function of Sms1: 1. Sms1 is involved in the BTB (Sert) and 2. Sms1 is involved in maintaining the integrity of syncytial elongating spermatid clusters (elSp). B) β-gal staining in epididymis indicates expression of Sms1 at the site of the BEB (PC; principal cells). β-gal staining was also detectable in epididymal spermatozoa (Sz).
(TIFF)

S1 Table Primary and secondary antibodies used for Western blotting (WB) and Immunohistochemistry (IHC). Primary and secondary antibodies are listed according to their immunogen. Dilutions of the antibodies are given for Western blotting (WB) and immunohistochemistry (IHC).
(XLSX)

S2 Table Differences between *Sms1* ^*WT*^ and mutant animals in respect to body and organ weights. Animals of the *Sms1* mouse line were weighed and dissected. Body weights, as well as weights of the organs are listed according to genotype and sex. Data is shown as mean ± SD.
(DOCX)

S3 Table Lipid profile testicular Cer species of *Sms1*^-/-^, compared to *Sms1*^*WT*^ levels. Levels of Cer species, sorted by intensities, are shown. Data is presented as mean percentage ± SEM, with *Sms1*^*WT*^ levels set to 100% (*Sms1*^*WT*^, n = 15; *Sms1*^-/-^, n = 17); *p≤0.05, **p≤0.01, ***p≤0.001.
(XLSX)
