# Supplementary figures and images for: Sphingomyelin Synthase 1 Is Essential for Male Fertility in Mice
Source: PLoS One. 2016 Oct 27;11(10):e0164298. doi: 10.1371/journal.pone.0164298 (PMC5082796; doi:10.1371/journal.pone.0164298)

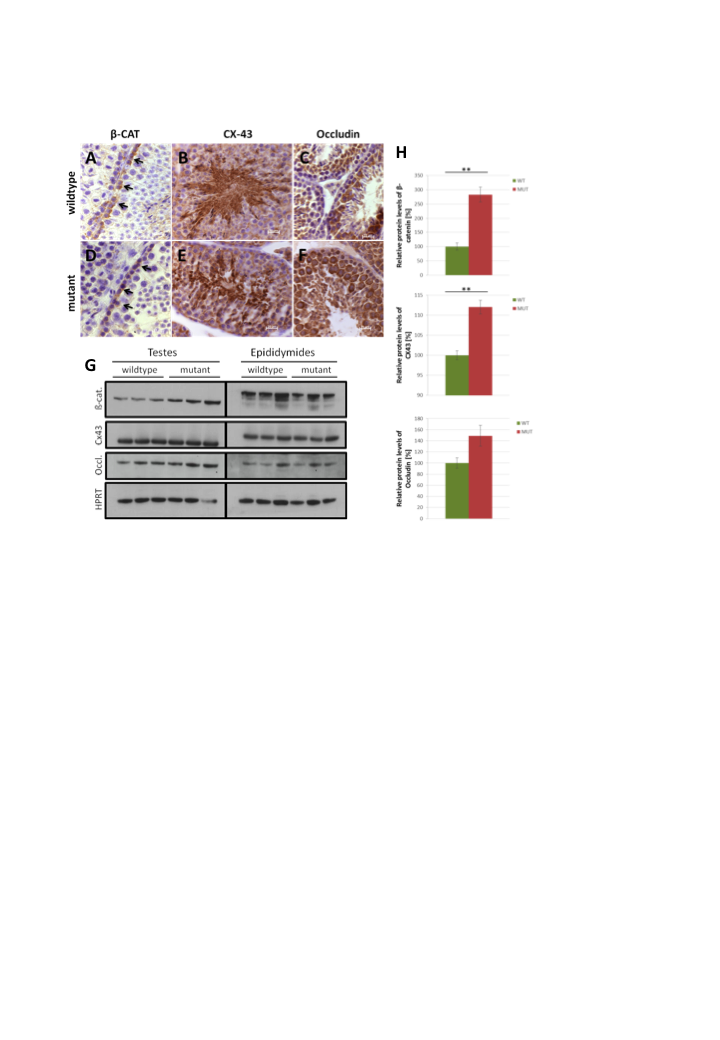

Supplement: S1 Fig — (A-F) Immunohistochemistry (IHC) for junctional marker proteins. (A, B, C) Sms1WT; (D, E, F) Sms1-/- animals (A, D) β-catenin (β-CAT) at the site of the BTB (black arrows). (B, E) Connexin-43 (CX-43). (C, F) Occludin. (G) Immunoblot for β-catenin, connexin-43 and occludin from testes and epididymides of Sms1WT and Sms1-/- males (n = 3 per genotype). (H) Quantification of β-catenin, connexin-43 and occludin testes protein levels. Sms1WT protein levels were set to 100%. Data is presented as mean percentage ± SEM (n = 3 per genotype); nsp≥0.05,*p≤0.05, **p≤0.01, ***p≤0.001. (TIFF) [file pone.0164298.s001.tiff]

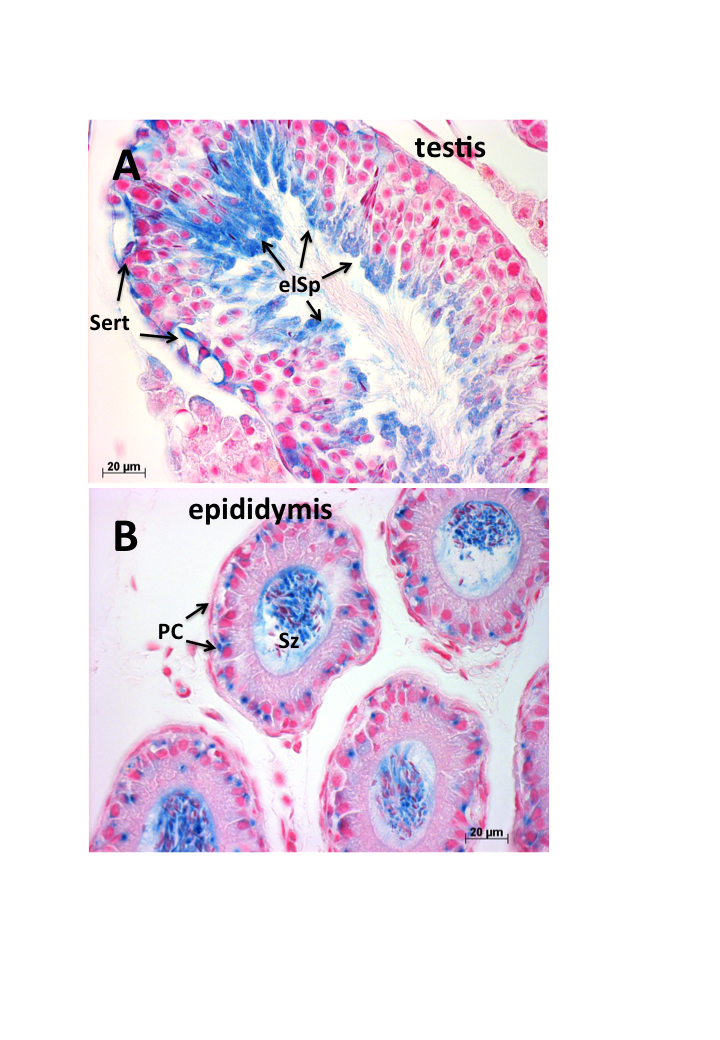

Supplement: S2 Fig — A) β-gal activity in testes of heterozygous males reflects wild-type expression of Sms1. The staining pattern indicates a dual function of Sms1: 1. Sms1 is involved in the BTB (Sert) and 2. Sms1 is involved in maintaining the integrity of syncytial elongating spermatid clusters (elSp). B) β-gal staining in epididymis indicates expression of Sms1 at the site of the BEB (PC; principal cells). β-gal staining was also detectable in epididymal spermatozoa (Sz). (TIFF) [file pone.0164298.s002.tiff]
