# Supplementary material for: Sphingomyelin Synthase 1 Is Essential for Male Fertility in Mice
Source: PLoS One. 2016 Oct 27;11(10):e0164298. doi: 10.1371/journal.pone.0164298 (PMC5082796; doi:10.1371/journal.pone.0164298)
Supplement: S2 Table — Animals of the Sms1 mouse line were weighed and dissected. Body weights, as well as weights of the organs are listed according to genotype and sex. Data is shown as mean ± SD. (DOCX) [file pone.0164298.s004.docx]

**S2 Table: Differences between *Sms1 ^WT^*** **and mutant animals in respect to body and organ weights.** Animals of the *Sms1* mouse line were weighed and dissected. Body weights, as well as weights of the organs are listed according to genotype and sex. Data is shown as mean ± SD.

| genotype | sex | body-weight (g)  mean ± SD | heart-weight (g)  mean ± SD | liver-weight (g)  mean ± SD | spleen-weight (g)  mean ± SD |
| --- | --- | --- | --- | --- | --- |
| mutant | f | 19.735  ± 1.410 | 0.104  ± 1.410 | 1.021  ± 1.410 | 0.074  ± 1.410 |
| wildtype | f | 21.528  ± 1.410 | 0.114  ± 0.006 | 1.053  ± 0.050 | 0.073  ± 0.007 |
| p-value |  | 0.133 | 0.155 | 0.610 | 0.910 |
| mutant | m | 24.779  ± 4.191 | 0.138  ± 0.023 | 1.098  ± 0.196 | 0.095  ± 0.048 |
| wildtype | m | 29.560  ± 4.488 | 0.147  ± 0.020 | 1.475  ± 0.253 | 0.079  ± 0.014 |
| p-value |  | 0.086 | 0.486 | 0.017 | 0.460 |
